# GCORE-sib: An efficient gene-gene interaction tool for genome-wide association studies based on discordant sib pairs

**DOI:** 10.1186/s12859-016-1145-z

**Published:** 2016-07-08

**Authors:** Pei-Yuan Sung, Yi-Ting Wang, Chao A. Hsiung, Ren-Hua Chung

**Affiliations:** Institute of Statistics, National Tsing Hua University, Hsin-Chu, Taiwan; Division of Biostatistics and Bioinformatics, Institute of Population Health Sciences, National Health Research Institutes, Zhunan, Taiwan

**Keywords:** Genome-wide association study, Gene-gene interaction, Discordant sib pair

## Abstract

**Background:**

A computationally efficient tool is required for a genome-wide gene-gene interaction analysis that tests an extremely large number of single-nucleotide polymorphism (SNP) interaction pairs in genome-wide association studies (GWAS). Current tools for GWAS interaction analysis are mainly developed for unrelated case-control samples. Relatively fewer tools for interaction analysis are available for complex disease studies with family-based design, and these tools tend to be computationally expensive.

**Results:**

We developed a fast gene-gene interaction test, GCORE-sib, for discordant sib pairs and implemented the test into an efficient tool. We used simulations to demonstrate that the GCORE-sib has correct type I error rates and has comparable power to that of the regression-based interaction test. We also showed that the GCORE-sib can run more than 10 times faster than the regression-based test. Finally, the GCORE-sib was applied to a GWAS dataset with approximately 2,000 discordant sib pairs, and the GCORE-sib finished testing 19,368,078,382 pairs of SNPs within 6 days.

**Conclusions:**

An efficient gene-gene interaction tool for discordant sib pairs was developed. It will be very useful for genome-wide gene-gene interaction analysis in GWAS using discordant sib pairs. The tool can be downloaded for free at http://gcore-sib.sourceforge.net.

**Electronic supplementary material:**

The online version of this article (doi:10.1186/s12859-016-1145-z) contains supplementary material, which is available to authorized users.

## Background

Genome-wide association studies (GWAS) are a popular strategy to investigate the genetic structure of complex diseases by identifying the association between single nucleotide polymorphisms (SNPs) and complex disorders. GWAS analysis is mainly focused on testing the effects of individual SNPs on complex diseases; however, complex diseases are likely to result from the interactions among multiple genes. That is, the presence of specific alleles in different genes can significantly increase the risk of developing a particular disease, such as Alzheimer’s disease, type 1 diabetes, autism, and schizophrenia [1–4]. In fact, most of the significant SNPs identified by GWAS can only explain a small proportion of the heritability of a disease. The missing heritability may be explained by gene-gene interactions [5]. Hence, the development of statistical gene-gene interaction tests based on GWAS has become important.

A computationally efficient test is required for a genome-wide interaction analysis that tests an extremely large number of SNP-SNP interaction pairs in GWAS (e.g., approximately 5 × 10^11^ interaction tests for a GWAS with 1 million SNPs). Several approaches, which can finish genome-wide interaction tests in a reasonable time while still maintaining statistical power, have been developed for GWAS with unrelated case-control samples. Some examples for these approaches include SNPHarvester [6], SNPRuler [7], and BOOST [8]. These approaches typically employ a two-stage analysis strategy; in the first stage, a rapid algorithm is used to identify a promising subset of SNPs with potential interaction effects, and in the second stage, a commonly used test such as the test based on logistic regression is used to identify pairwise interactions from the subset of SNPs.

Current interaction tests for family-based studies are computationally intensive, which prevent the applications of the tests to genome-wide interaction analysis. For example, MDR-PDT [9] and PGMDR [10] are extended from the machine learning-based Multifactor Dimensionality Reduction (MDR) test [11], which involves intensive calculations such as cross-validations and permutations. Regression-based tests such as conditional logistic regression (CLR) and generalized estimating equations (GEE) [12] can also be used for testing interactions [13]; however, iterative algorithms such as the Newton-Raphson method are required to estimate the parameters. As many family-based GWAS have been conducted [14–17], it becomes important to develop a computationally efficient interaction test for family-based GWAS.

To overcome the computational challenges in current family-based interaction tests, here we developed an efficient gene-gene interaction test for discordant sib pairs (DSPs), the GCORE-sib, which takes into consideration the correlations in DSPs, and implemented the test into an efficient tool for family-based interaction analysis. The GCORE-sib is extended from the fast epistasis statistic implemented in PLINK [18], which is an odds ratio-based interaction test for case-control studies [19]. The log odds ratios, which measure the correlations between two SNPs, are first calculated for affected and unaffected siblings, and the difference in the log odds ratios is compared in the GCORE-sib statistic. Variance and covariance for the statistic were calculated based on appropriate theoretical models, and the distribution of the statistic was assumed to follow a standard normal distribution. Therefore, the statistic and its p-value were rapidly calculated. We used simulation studies to evaluate the type I error rates for the GCORE-sib test, and to compare the power of the test with that of GEE and MDR-PDT. The GCORE-sib software was implemented with POSIX threads (Pthreads), which allow for parallel computing of the SNP pairs. We compared the performance in terms of run time among the GCORE-sib, GEE, and MDR-PDT. Finally, a GWAS dataset was used to evaluate the run time of the GCORE-sib in the genome-wide scale.

## Implementation

### The GCORE-sib statistic

The GCORE-sib statistic was developed from the PLINK interaction statistic [18] and is calculated based on the difference in log odds ratios between cases and controls in families. In the test, we considered discordant sib pair in each nuclear family (DSP; one affected and one unaffected sib). Affected and unaffected sibs are defined as cases and controls, respectively. Assume we have *k* independent discordant sib pairs. Let *n*_*ij*_ be the number of affected sibs with genotypes *i* and *j* at the two SNPs *M*_1_ and *M*_2_, where *i* = 1, 2, 3 (for genotypes *AA*, *Aa*, and *aa*, respectively) and *j* = 1, 2, 3 (for genotypes *BB*, *Bb*, and *bb*, respectively). Suppose that in the *k* discordant sib pairs, *R*_*ij*_ is the number of sibs with genotypes *i* and *j* at the two SNPs (including the affected and unaffected sibs). Therefore, we can construct the genotype tables for the affected and unaffected sibs, as shown in Tables 1 and 2. Each cell count in Table 1 represents the total number of affected sibs with a specific genotype in all *k* discordant sib pairs. That is, *n*_*ij*_ = ∑_*s* = 1_^*k*^*n*_*ij*_^*s*^ for *i* = 1, 2, 3 and *j* = 1, 2, 3, where *n*_*ij*_^*s*^ represents the number of affected sibs with genotypes *i* and *j* in *s*^th^ discordant sib pair. Similar to S-TDT [20], we assumed that the random variables (*N*_11_^*s*^, *N*_12_^*s*^, …, *N*_33_^*s*^) with the observed values of (*n*_11_^*s*^, *n*_12_^*s*^, …, *n*_33_^*s*^) follow a multivariate hypergeometric distribution.

**Table 1:** Counts of genotypes in the affected sibs in the *k* discordant sib pairs

	*AA*	*Aa*	*aa*
*BB*	*n*_11_	*n*_12_	*n*_13_
*Bb*	*n*_21_	*n*_22_	*n*_23_
*bb*	*n*_31_	*n*_32_	*n*_33_

**Table 2:** Counts of genotypes in the unaffected sibs in the *k* discordant sib pairs

	*AA*	*Aa*	*aa*
*BB*	*R*_11_ − *n*_11_	*R*_12_ − *n*_12_	*R*_13_ − *n*_13_
*Bb*	*R*_21_ − *n*_21_	*R*_22_ − *n*_22_	*R*_23_ − *n*_23_
*bb*	*R*_31_ − *n*_31_	*R*_32_ − *n*_32_	*R*_33_ − *n*_33_

We followed the same procedure in PLINK to collapse the pair of 3 × 3 genotype tables into a pair of 2 × 2 tables for cases and controls as shown in Tables 3 and 4. According to Tables 3 and 4, the odds ratios between SNPs *M*_1_ and *M*_2_ for cases and controls can be calculated as:

**Table 3:** Counts of alleles in the affected sibs

	Case	
	*A*	*a*
*B*	4*n*_11_ + 2*n*_12_ + 2*n*_21_ + *n*_22_	4*n*_13_ + 2*n*_12_ + 2*n*_23_ + *n*_22_
*b*	4*n*_31_ + 2*n*_32_ + 2*n*_21_ + *n*_22_	4*n*_33_ + 2*n*_32_ + 2*n*_23_ + *n*_22_

**Table 4:** Counts of alleles in the unaffected sibs

	Control	
	*A*	*a*
*B*	$$ \begin{array}{l}4\left({R}_{11}-{n}_{11}\right)+2\left({R}_{12}-{n}_{12}\right)\\ {}+2\left({R}_{21}-{n}_{21}\right)+\left({R}_{22}-{n}_{22}\right)\end{array} $$	$$ \begin{array}{l}4\left({R}_{13}-{n}_{13}\right)+2\left({R}_{12}-{n}_{12}\right)\\ {}+2\left({R}_{23}-{n}_{23}\right)+\left({R}_{22}-{n}_{22}\right)\end{array} $$
*b*	$$ \begin{array}{l}4\left({R}_{31}-{n}_{31}\right)+2\left({R}_{32}-{n}_{32}\right)\\ {}+2\left({R}_{21}-{n}_{21}\right)+\left({R}_{22}-{n}_{22}\right)\end{array} $$	$$ \begin{array}{l}4\left({R}_{33}-{n}_{33}\right)+2\left({R}_{32}-{n}_{32}\right)\\ {}+2\left({R}_{23}-{n}_{23}\right)+\left({R}_{22}-{n}_{22}\right)\end{array} $$

1 $$ O{R}_{case}=\frac{\left(4{n}_{11}+2{n}_{12}+2{n}_{21}+{n}_{22}\right)\left(4{n}_{33}+2{n}_{32}+2{n}_{23}+{n}_{22}\right)}{\left(4{n}_{13}+2{n}_{12}+2{n}_{23}+{n}_{22}\right)\left(4{n}_{31}+2{n}_{32}+2{n}_{21}+{n}_{22}\right)} $$

2 $$ \begin{array}{l}O{R}_{control}=\frac{\left[4\left({R}_{11}-{n}_{11}\right)+2\left({R}_{12}-{n}_{12}\right)+2\left({R}_{21}-{n}_{21}\right)+\left({R}_{22}-{n}_{22}\right)\right]}{\left[4\left({R}_{13}-{n}_{13}\right)+2\left({R}_{12}-{n}_{12}\right)+2\left({R}_{23}-{n}_{23}\right)+\left({R}_{22}-{n}_{22}\right)\right]}\\ {}\kern5.12em \times \frac{\left[4\left({R}_{33}-{n}_{33}\right)+2\left({R}_{32}-{n}_{32}\right)+2\left({R}_{23}-{n}_{23}\right)+\left({R}_{22}-{n}_{22}\right)\right]}{\left[4\left({R}_{31}-{n}_{31}\right)+2\left({R}_{32}-{n}_{32}\right)+2\left({R}_{21}-{n}_{21}\right)+\left({R}_{22}-{n}_{22}\right)\right]}\end{array} $$

Similar to the PLINK approach, under the assumptions of Hardy-Weinberg Equilibrium (HWE) and Linkage Equilibrium (LE) for the two SNPs, the GCORE-sib statistic for the gene-gene interaction test can be constructed based on a Z-score as follows.

3 $$ {G}_{DSP}=\frac{\left[ log\left({\widehat{OR}}_{case}\right)- log\left({\widehat{OR}}_{control}\right)\right]}{\sqrt{\widehat{Var}\left[ log\left({\widehat{OR}}_{case}\right)- log\left({\widehat{OR}}_{control}\right)\right]}} $$

where ${\widehat{OR}}_{case}$ and ${\widehat{OR}}_{control}$ are the sample estimators for *OR*_*case*_ and *OR*_*control*_, respectively. The null hypothesis of the GCORE-sib test is that the two SNPs tested do not have interaction effects on the disease.

Due to the correlation of genotypes between discordant sibs, the covariance between the two log odds ratios needs to be considered. Based on the multivariate hypergeometric distribution assumption, we can calculate the variance and covariance for the two odds ratios. The detailed derivation is shown in Additional file 1. Based on the derivation, the covariance is calculated as follows

4 $$ \mathrm{C}\mathrm{o}\mathrm{v}\left( log\left({\widehat{OR}}_{case}\right), log\left({\widehat{OR}}_{control}\right)\right)=-Var\left( log\left({\widehat{OR}}_{case}\right)\right)=-Var\left( log\left({\widehat{OR}}_{control}\right)\right) $$

Therefore, the GCORE-sib statistic can be written as

5 $$ {G}_{DSP}=\frac{\left[ log\left({\widehat{OR}}_{case}\right)- log\left({\widehat{OR}}_{control}\right)\right]}{\sqrt{4\widehat{Var}\left( log\left({\widehat{OR}}_{case}\right)\right)}} $$

The calculation of $\widehat{Var}\left( log\left({\widehat{OR}}_{case}\right)\right)$ is also shown in Additional file 1.

### Simulations

We used the Sequence and phenotype Simulator, SeqSIMLA [21], to evaluate the type I error rates for the GCORE-sib and to compare the power of the GCORE-sib with other methods under different scenarios. SeqSIMLA requires of a population of sequences generated by other programs. Therefore, we downloaded the haplotypes for the Han Chinese population (CHB) in the HapMap3 project as a reference panel. Then we used the HAPGEN version 2 (HAPGEN2) [22] to produce simulated haplotypes based on the reference panel. HAPGEN2 can simulate haplotypes with similar LD structures and allele frequencies to that of the reference panel. We randomly selected two genes that were not linked as the simulated region and generated a total of 10,000 haplotypes in the two genes. Based on the 10,000 haplotypes, SeqSIMLA first simulated haplotypes in founders and assumed random mating to generate the offspring. We chose the logistic function as the penetrance function in SeqSIMLA:

$$ \mathrm{P}\left(\mathrm{Affected}\Big|\boldsymbol{X}\right) = \frac{ \exp\;\left(\rho +{\lambda}_1{X}_1+{\lambda}_2{X}_2+{\lambda}_3{X}_1{X}_2\right)}{1 + \exp \left(\rho +{\lambda}_1{X}_1+{\lambda}_2{X}_2+{\lambda}_3{X}_1{X}_2\right)}, $$

where ***X*** = (*X*_*1*_,*X*_*2*_) is a vector of genotype coding based on additive, dominant, or recessive model for the two disease SNPs; *ρ* is the parameter used to determine the disease prevalence; *λ*_1_ and *λ*_2_ represent the effect sizes of the main effects for the disease SNPs; and *λ*_3_ determines the interaction effect for the two disease SNPs.

For the type I error simulations, we first simulated no interaction effects and no main effects for two SNPs in the two genes. Different minor allele frequencies (MAFs) at the two SNPs (i.e., (0.2, 0.2; 0.3, 0.15)) and different numbers of DSPs (i.e., 250, 500, and 1000) were considered. We then considered the situation where main effects were present for the two SNPs under different levels of disease prevalence (i.e., 1 %, 5 %, and 10 %). We simulated one scenario where only one SNP had main effect (i.e., *λ*_1_ = log (2), *λ*_2_ = 0) and three scenarios where both SNPs had main effects (i.e., *λ*_1_ = log (1.3), *λ*_2_ = log (1.3); *λ*_1_ = log (1.5), *λ*_2_ = log (1.5); *λ*_1_ = log (2), *λ*_2_ = log (2)). In addition, to investigate whether the GCORE-sib is robust to the violation of the assumption of LE, we simulated two SNPs in different levels of LD (i.e., LD measures *r*^2^ = 0.3, 0.5, and 0.8). All type I error rates in these scenarios were calculated based on 5,000 replicates of samples. Two significance levels (i.e., 0.05 and 0.01) were considered for the type I error calculations.

For the power studies, we simulated interaction effects for two SNPs in the two genes. We compared the power of our method with the power for GEE and MDR-PDT under different scenarios. The “exchangeable” within cluster correlation structure was specified in GEE. The regression model based on GEE included individual terms for the two SNPs and one interaction term, where genotypes were coded as the minor allele counts. We considered different numbers of DSPs (i.e., 250, 500, and 1000), disease models (i.e., additive, dominant, and recessive), MAFs (i.e., 0.2, 0.2; 0.3, 0.15), and effect sizes (i.e., *λ*_1_ = 0, *λ*_2_ = 0, *λ*_3_ = log (2); *λ*_1_ = 0, *λ*_2_ = 0, *λ*_3_ = log (2.25)). The default settings were 500 DSPs, additive model, MAFs of 0.2 for the two SNPs, and effect size of (*λ*_1_ = 0, *λ*_2_ = 0, *λ*_3_ = log (2)). We changed one parameter at a time for each of the scenarios. Table 5 shows the parameter values for each of the scenarios. The power was calculated with a significance level of 0.05 based on 1,000 replicates of samples.

**Table 5:** Parameter settings for the power simulations

Scenario	Parameters (NF, DM, MAF, ES)^a^
Scen1	NF: 250,500,1000; DM: Additive; MAF: (0.2,0.2); ES: log(2)
Scen2	NF: 500; DM: Additive, Dominant, Recessive; MAF: (0.2,0.2); ES: log(2)
Scen3	NF: 500; DM: Additive; MAF: (0.2,0.2),(0.3,0.15); ES: log(2)
Scen4	NF: 500; DM: Additive; MAF: (0.2,0.2); ES: log(2), log(2.25)

^a^ *NF* number of families, *DM* disease model, *MAF* minor allele frequencies for the two SNPs, *ES* effect size for the interaction

### Parallel computing

Although the calculation of the GCORE-sib statistic is fast for a SNP pair, performing a genome-wide interaction analysis by testing tens of billions of tests can still be very time consuming for the GCORE-sib. The GCORE-sib software was implemented with POSIX Threads (Pthreads) so that the calculations for SNP pairs can be performed in parallel on a multi-core computer. Moreover, the calculations can be performed for SNPs between a chromosome pair specified by the user. Therefore, the calculations can be distributed across different computers.

### Performance comparison

We compared the performance of the GCORE-sib with GEE and MDR-PDT. Currently, GEE is mostly implemented in R, which is not comparable to the GCORE-sib and MDR-PDT implemented in C++. Alternatively, we used the interaction test based on a regular logistic regression implemented in PLINK. The logistic regression based on GEE usually first runs the regular logistic regression to obtain initial parameter estimates assuming all samples are independent, and more iterations are performed for the overall parameter estimates including the correlation matrix. Therefore, the logistic regression with GEE is expected to run longer than the regular logistic regression. A total of 1,000 DSPs were simulated using SeqSIMLA, and the performance was compared based on 1,000, 5,000, and 10,000 pairs of SNPs on a computer with Xeon 2.0 GHz CPU and 96 GB of RAM.

To evaluate the performance of the GCORE-sib for a genome-wide interaction analysis, we downloaded the Wellcome Trust Case Control Consortium (WTCCC) GWAS dataset for hypertension. The dataset consists of 1,952 unrelated cases for hypertension and 2,938 unrelated controls, and there are 457,710 SNPs in the data. We randomly matched cases and controls, which resulted in 1,952 case-control pairs. The case-control pairs were analyzed as DSPs in the GCORE-sib. Because the WTCCC study is not a family-based study, our analysis was used only for the performance evaluation for the GCORE-sib. We also downloaded the gene annotation file from the UCSC genome browser website. All possible pairs of SNP interactions between genes were tested in parallel with 20 cores by the GCORE-sib on the aforementioned computer.

## Results

### Type I error rates

Table 6 shows the type I error estimates for the GCORE-sib under different MAFs at the two SNPs and different numbers of DSPs at the significance levels of 0.05 and 0.01. The type I error rates were close to the nominal levels under all scenarios. Table 7 summarizes the results of the type I error rates in the presence of main effects. In the presence of only one main effect (i.e., *λ*_1_ = log (2), *λ*_2_ = 0), the type I error estimates were close to the 0.05 nominal level across different levels of disease prevalence and disease models. The type I error estimates were inflated by the large effect size (e.g., *λ*_1_ = log (2), *λ*_2_ = log (2)) when both SNPs had main effects. When there was LD between the two SNPs, the type I error rates were 0.046, 0.052, and 0.054 for LD *r*^*2*^ of 0.3, 0.5, and 0.8, respectively, at the significance level of 0.05, and the type I error rates were 0.0082, 0.0088, and 0.0104 for LD *r*^*2*^ of 0.3, 0.5, and 0.8, respectively, at the significance level of 0.01. Therefore, the GCORE-sib also maintained proper type I error rates when the assumption of LE was violated.

**Table 6:** Type I error rate simulations for two SNPs with MAFs of (0.2 and 0.2; 0.3 and 0.15) and with different numbers of DSPs at the significant levels of 0.05 and 0.01

MAF/number of DSPs	α = 0.05	α = 0.01
MAF 0.2, 0.2		
250 DSPs	0.0486	0.0092
500 DSPs	0.0494	0.0086
1000 DSPs	0.0500	0.0106
MAF 0.3, 0.15		
250 DSPs	0.0502	0.0114
500 DSPs	0.0484	0.0106
1000 DSPs	0.0510	0.0124

**Table 7:** Type I error rates for different disease models, main effects, and disease prevalences

		Disease prevalence		
Effect size	Disease model	0.01	0.05	0.1
*λ*_1_ = log (2), *λ*_2_ = 0	Additive	0.0484	0.0534	0.0488
	Dominant	0.0544	0.0502	0.0560
	Recessive	0.0494	0.0440	0.0454
*λ*_1_ = log (1.3), *λ*_2_ = log (1.3)	Additive	0.0476	0.0530	0.0458
	Dominant	0.0524	**0.0570** ^**a**^	0.0530
	Recessive	0.0474	0.0532	0.0522
*λ*_1_ = log (1.5), *λ*_2_ = log (1.5)	Additive	0.0534	0.0500	0.0508
	Dominant	**0.0674**	**0.0674**	**0.0612**
	Recessive	0.0518	0.0520	0.0498
*λ*_1_ = log (2), *λ*_2_ = log (2)	Additive	**0.0768**	**0.0658**	**0.0602**
	Dominant	**0.1670**	**0.1268**	**0.0764**
	Recessive	0.0448	0.0514	0.0520

^a^Values in bold represent that the 95 % confidence intervals of the estimates do not include the expected level of 0.05

### Power

**Fig. 1:**
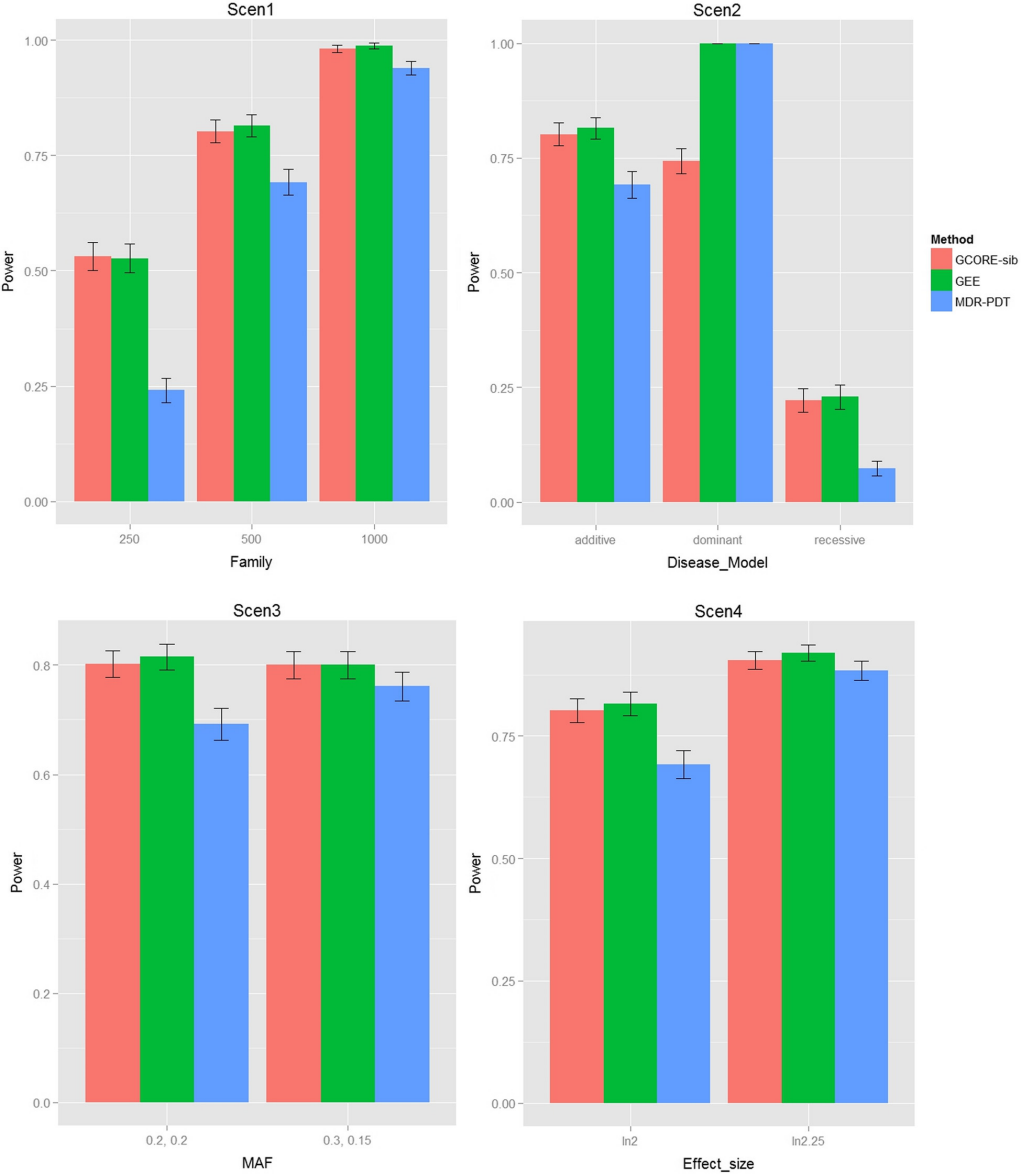
Power comparison for GCORE-sib, GEE, and MDR-PDT under Scen1-4 described in Table 5. The error bars represent the 95 % confidence intervals for the power

Figure 1 shows the power comparisons under different scenarios. In Scen1, the power for the GCORE-sib was similar to the power for GEE, while MDR-PDT had the lowest power. For all different methods, the power increased with the increase in the number of DSPs. In Scen2, for the additive and recessive models, similar power patterns were observed that the power for the GCORE-sib and GEE was similar, and the power for both tests was higher than the power for MDR-PDT. However, in the dominant model, GEE and MDR-PDT can have significantly higher power than that for the GCORE-sib. The GCORE-sib had the highest power in the additive model, followed by the dominant and recessive models, while the other tests showed the highest power in the dominant model, followed by the additive and recessive models. In Scen3, the power for the GCORE-sib and GEE in MAF of (0.2, 0.2) and MAF of (0.3, 015) was similar, while MDR-PDT showed higher power in MAF of (0.3, 0.15) than that in MAF of (0.2, 0.2). In the last scenario where the interaction effect size is increased to log(2.25), the power for the GCORE-sib was still close to the power for GEE, and the power for both tests was also higher than the power for MDR-PDT. In summary, the power for the GCORE-sib was generally similar to the power for GEE and the power for the GCORE-sib and GEE was generally higher than the power for MDR-PDT under the additive model in our simulations.

### Performance comparison

Table 8 shows the run time for the GCORE-sib, PLINK, and MDR-PDT for testing 1,000, 5,000, and 10,000 pairs of SNPs in 1,000 DSPs. The GCORE-sib finished testing these SNP pairs in 2 seconds, while PLINK implementing the regular logistic regression required more than 10 times of the run time for testing the same number of pairs of SNPs as the GCORE-sib. The logistic regression based on GEE would require more time than the regular logistic regression. For example, using the gee package in R (via the gee() function) for the interaction test in logistic regression requires approximately 13 times of the run time compared to the regular logistic regression in R (via the glm() function), based on the same model and the same samples we used for PLINK. Therefore, the GCORE-sib can potentially run 100 times faster than the logistic regression based on GEE when implemented in C++. Moreover, MDR-PDT spent significantly more time than the GCORE-sib and PLINK. For example, MDR-PDT required 6 hours and 52 minutes to test the 10,000 pairs of SNPs, when compared to 2 seconds and 26 seconds for the GCORE-sib and PLINK, respectively. On the other hand, the GCORE-sib spent 5 days and 12 hours on testing 19,368,078,382 pairs of SNPs in the WTCCC GWAS dataset. Therefore, the GCORE-sib can finish a genome-wide interaction analysis in a reasonable time frame.

**Table 8:** Performance comparison for the GCORE-sib, PLINK, and MDR-PDT

SNP pairs	GCORE-sib	PLINK	MDR-PDT
1,000	0.2 s	3 s	43 m 57 s
5,000	1.4 s	16 s	3 h 29 m 32 s
10,000	2 s	26 s	6 h 52 m 36 s

## Discussion

We developed an odds ratio-based gene-gene interaction test considering correlations in discordant sib pairs. The hypergeometric distribution for genotype counts was assumed in each discordant sib pair. Then the estimates of correlation within families can be calculated based on the model assumption. We demonstrated that the GCORE-sib showed appropriate type I error rates under the null hypothesis of no interaction, even in the presence of LD between SNPs, or when only one SNP showed main effect. Sharing the same property as the odds ratio-based test for case-control studies, the GCORE-sib maintains proper type I error rates when only one SNP has main effects. When the two SNPs both have strong main effects, type I error rates could be inflated for most of the interaction tests [23]. Therefore, in practice, significant results from interaction tests for two SNPs should be interpreted along with tests for main effects for the same two SNPs.

We also compared the power of the GCORE-sib with two alternative family-based interaction tests, GEE and MDR-PDT. Our simulation results suggested that the GCORE-sib and GEE had similar power, while MDR-PDT had the lowest power under most of the simulation scenarios. Under the assumption of HWE, alleles are independent in Tables 3 and 4, and the GCORE-sib tests the allelic correlations between the two SNPs based on the two tables. Hence, an additive model is implicitly assumed in the GCORE-sib. Moreover, genotypes for GEE were also coded based on an additive model in our simulations. As most of our power simulations were conducted under the additive model, it was not surprising to observe higher power for the GCORE-sib and GEE than the machine learning-based MDR-PDT.

GEE and MDR-PDT are not suitable for genome-wide interaction tests, due to the high computational burden. In contrast, the GCORE-sib is demonstrated to be able to perform a rapid test for each pair of SNP-SNP interactions. However, GEE is flexible of incorporating covariates in the test. Therefore, the GCORE-sib can be used as a complementary tool to GEE for analyzing DSPs. That is, the GCORE-sib can be used as a screening tool to identify candidate SNP pairs with interactions. Then GEE can be used to test for interactions for the candidate SNP pairs by incorporating appropriate covariates.

## Conclusions

In conclusion, we have developed an efficient gene-gene interaction test for DSPs, which is suitable for genome-wide interaction analysis for SNP pairs in DSPs. We have implemented the method in C++, which can be downloaded for free at http://gcore-sib.sourceforge.net.

## Additional file

Additional file 1: Derivation for the variance of the GCORE statistic (DOCX 94 kb)
