# Supplementary material for: GCORE-sib: An efficient gene-gene interaction tool for genome-wide association studies based on discordant sib pairs
Source: BMC Bioinformatics. 2016 Jul 8;17:273. doi: 10.1186/s12859-016-1145-z (PMC4939061; doi:10.1186/s12859-016-1145-z)
Supplement: Additional file 1: — Derivation for the variance of the GCORE statistic (DOCX 94 kb) [file 12859_2016_1145_MOESM1_ESM.docx]

**Derivation for the variance of the GCORE statistic**

Let $N_{11}$, $N_{12}$, … , $N_{33}$ be the random variables for the cell counts from Table 1.

We denote the logarithms of the two ORs in equations (1) and (2) as $\hat{\beta}_{1}$ and $\hat{\beta}_{2}$, respectively. Then, the estimators of the two log-odds ratios are given by

$$\widehat{\beta}_{1}=\log\widehat{OR}_{case}=\log\left[\frac{(4N_{11}+2N_{12}+2N_{21}+N_{22})(4N_{33}+2N_{32}+2N_{23}+N_{22})}{(4N_{13}+2N_{12}+2N_{23}+N_{22})(4N_{31}+2N_{32}+2N_{21}+N_{22})}\right]$$

(A1)

$$\begin{matrix} \widehat{\beta}_{2}=\log\widehat{OR}_{control}= \\ \log\left\{\begin{matrix} \frac{\left[4\left(R_{11}-N_{11}\right)+2\left(R_{12}-N_{12}\right)+2\left(R_{21}-N_{21}\right)+\left(R_{22}-N_{22}\right)\right]}{\left[4\left(R_{13}-N_{13}\right)+2\left(R_{12}-N_{12}\right)+2\left(R_{23}-N_{23}\right)+\left(R_{22}-N_{22}\right)\right]}\times\\ \frac{\left[4\left(R_{33}-N_{33}\right)+2\left(R_{32}-N_{32}\right)+2\left(R_{23}-N_{23}\right)+\left(R_{22}-N_{22}\right)\right]}{\left[4\left(R_{31}-N_{31}\right)+2\left(R_{32}-N_{32}\right)+2\left(R_{21}-N_{21}\right)+\left(R_{22}-N_{22}\right)\right]} \end{matrix}\right\} \end{matrix}$$

(A2)

The variance for the difference in $\hat{\beta}_{1}$ and $\hat{\beta}_{2}$ is calculated as:

(A3)

First, we calculate $\text{Cov}\left(\widehat{\beta}_{1},\text{ }\widehat{\beta}_{1}\right)$ in Equation (A3)

$$\begin{matrix} \text{Cov}\left(\widehat{\beta}_{1},\text{ }\widehat{\beta}_{1}\right) \\ =\text{Cov}\left\{\log\left[\frac{(4N_{11}+2N_{12}+2N_{21}+N_{22})(4N_{33}+2N_{32}+2N_{23}+N_{22})}{(4N_{13}+2N_{12}+2N_{23}+N_{22})(4N_{31}+2N_{32}+2N_{21}+N_{22})}\right],\text{ }\right. \\ \left.\text{ }\log\left[\frac{(4N_{11}+2N_{12}+2N_{21}+N_{22})(4N_{33}+2N_{32}+2N_{23}+N_{22})}{(4N_{13}+2N_{12}+2N_{23}+N_{22})(4N_{31}+2N_{32}+2N_{21}+N_{22})}\right]\right\} \\ =\text{Cov}\left[\log\right.(4N_{11}+2N_{12}+2N_{21}+N_{22})+\log(4N_{33}+2N_{32}+2N_{23}+N_{22})- \\ \text{ log}(4N_{13}+2N_{12}+2N_{23}+N_{22})-\log(4N_{31}+2N_{32}+2N_{21}+N_{22}),\text{ } \\ \text{ log}(4N_{11}+2N_{12}+2N_{21}+N_{22})+\log(4N_{33}+2N_{32}+2N_{23}+N_{22})- \\ \text{ }\left.\text{log}(4N_{13}+2N_{12}+2N_{23}+N_{22})-\log(4N_{31}+2N_{32}+2N_{21}+N_{22})\right] \end{matrix}$$

(A4)

We can approximate the above covariance using the Delta method with a first-order Taylor expansion around the means. For example, we calculate the first item in the expansion of Equation (A4)

$\begin{matrix} \text{Cov}\left[\log(4N_{11}+2N_{12}+2N_{21}+N_{22}),\text{ log(4}N_{11}+2N_{12}+2N_{21}+N_{22})\right] \\ \approx\frac{1}{4\text{E}(N_{11})+2\text{E(}N_{12})+2\text{E(}N_{21})+\text{E(}N_{22})}\frac{1}{4\text{E}(N_{11})+2\text{E(}N_{12})+2\text{E(}N_{21})+\text{E(}N_{22})}\times\\ \text{ Cov(}4N_{11}+2N_{12}+2N_{21}+N_{22},\text{ }4N_{11}+2N_{12}+2N_{21}+N_{22}) \\ =\frac{1}{4\text{E}\left(\sum_{s=1}^{k} N_{11}^{s}\right)+2\text{E}\left(\sum_{s=1}^{k} N_{12}^{s}\right)+2\text{E}\left(\sum_{s=1}^{k} N_{21}^{s}\right)+\text{E}\left(\sum_{s=1}^{k} N_{22}^{s}\right)}\times\\ \text{ }\frac{1}{4\text{E}\left(\sum_{s=1}^{k} N_{11}^{s}\right)+2\text{E}\left(\sum_{s=1}^{k} N_{12}^{s}\right)+2\text{E}\left(\sum_{s=1}^{k} N_{21}^{s}\right)+\text{E}\left(\sum_{s=1}^{k} N_{22}^{s}\right)}\times\\ \text{ Cov}\left(4\sum_{s=1}^{k} N_{11}^{s}+2\sum_{s=1}^{k} N_{12}^{s}+2\sum_{s=1}^{k} N_{21}^{s}+\sum_{s=1}^{k} N_{22}^{s},\text{ }4\sum_{s=1}^{k} N_{11}^{s}+2\sum_{s=1}^{k} N_{12}^{s}+2\sum_{s=1}^{k} N_{21}^{s}+\sum_{s=1}^{k} N_{22}^{s}\right) \end{matrix}$(A5)

Because $N_{ij}^{s}$ and $N_{hg}^{t}$ are independent for all *i, j, h, g* while *s$\neq$t*, A5 can be written as

$\begin{matrix} \left\{\frac{1}{\sum_{s=1}^{k} \left[4\text{E(}N_{11}^{s})+2\text{E(}N_{12}^{s})+2\text{E(}N_{21}^{s})+\text{E(}N_{22}^{s})\right]}\right\}\left\{\frac{1}{\sum_{s=1}^{k} \left[4\text{E(}N_{11}^{s})+2\text{E(}N_{12}^{s})+2\text{E(}N_{21}^{s})+\text{E(}N_{22}^{s})\right]}\right\}\times\\ \text{ }\sum_{s=1}^{k} \left[\text{Cov(4}N_{11}^{s}+2N_{12}^{s}+2N_{21}^{s}+N_{22}^{s},\text{ 4}N_{11}^{s}+2N_{12}^{s}+2N_{21}^{s}+N_{22}^{s}\right] \end{matrix}$(A6)

Assume $(N_{11}^{s}, N_{12}^{s}, \ldots, N_{33}^{s})$ $\sim$ Multivariate Hypergeometric (2, $R_{11}^{s}$*,$R_{12}^{s}$ , …,* $R_{44}^{s}$, 1), as described in Methods. Based on the multivariate hypergeometric distribution, we have

$$\text{E(}N_{ij}^{s})=\frac{R_{ij}^{s}}{2}\text{ };\text{ Var(}N_{ij}^{s})=\frac{R_{ij}^{s}}{2}(1-\frac{R_{ij}^{s}}{2})\text{ };\text{ Cov(}N_{ij}^{s},\text{ }N_{hg}^{s})=-\frac{R_{ij}^{s}}{2}\frac{R_{hg}^{s}}{2}$$

,where $\sum_{s=1}^{k} R_{ij}^{s}=R_{ij}\text{ for all }i,\text{ }j,\text{ }h\neq i,\text{ }g\neq j.$

Thus,

$$\begin{matrix} \text{Cov}\left[\log(4N_{11}+2N_{12}+2N_{21}+N_{22}),\text{ log(4}N_{11}+2N_{12}+2N_{21}+N_{22})\right] \\ =\frac{1}{\left(2R_{11}+R_{12}+R_{21}+\frac{R_{22}}{2}\right)}\frac{1}{\left(2R_{11}+R_{12}+R_{21}+\frac{R_{22}}{2}\right)}\times\\ \text{ }\sum_{s=1}^{k} \left[\left(8R_{11}^{s}+2R_{12}^{s}+2R_{21}^{s}+\frac{R_{22}^{s}}{2}\right)-\left(2R_{11}^{s}+R_{12}^{s}+R_{21}^{s}+\frac{R_{22}^{s}}{2}\right)^{2}\right] \end{matrix}$$

(A7)

We can use the same derivation as shown in Equation (A7) to calculate all items in Equation (A4).

$$\begin{matrix} \text{Cov}\left(\widehat{\beta}_{1},\text{ }\widehat{\beta}_{1}\right)\approx\\ \frac{\sum_{s=1}^{k} \left[\left(8R_{11}^{s}+2R_{12}^{s}+2R_{21}^{s}+\frac{R_{22}^{s}}{2}\right)-\left(2R_{11}^{s}+R_{12}^{s}+R_{21}^{s}+\frac{R_{22}^{s}}{2}\right)^{2}\right]}{\left(2R_{11}+R_{12}+R_{21}+\frac{R_{22}}{2}\right)^{2}}+ \\ \frac{\sum_{s=1}^{k} \left[\left(8R_{33}^{s}+2R_{32}^{s}+2R_{23}^{s}+\frac{R_{22}^{s}}{2}\right)-\left(2R_{33}^{s}+R_{32}^{s}+R_{23}^{s}+\frac{R_{22}^{s}}{2}\right)^{2}\right]}{\left(2R_{33}+R_{32}+R_{23}+\frac{R_{22}}{2}\right)^{2}}+ \\ \frac{\sum_{s=1}^{k} \left[\left(8R_{13}^{s}+2R_{12}^{s}+2R_{23}^{s}+\frac{R_{22}^{s}}{2}\right)-\left(2R_{13}^{s}+R_{12}^{s}+R_{23}^{s}+\frac{R_{22}^{s}}{2}\right)^{2}\right]}{\left(2R_{13}+R_{12}+R_{23}+\frac{R_{22}}{2}\right)^{2}}+ \\ \frac{\sum_{s=1}^{k} \left[\left(8R_{31}^{s}+2R_{32}^{s}+2R_{21}^{s}+\frac{R_{22}^{s}}{2}\right)-\left(2R_{31}^{s}+R_{32}^{s}+R_{21}^{s}+\frac{R_{22}^{s}}{2}\right)^{2}\right]}{\left(2R_{31}+R_{32}+R_{21}+\frac{R_{22}}{2}\right)^{2}}+ \\ 2\frac{\sum_{s=1}^{k} \left[\left(\frac{R_{22}^{s}}{2}\right)-\left(2R_{11}^{s}+2R_{12}^{s}+2R_{21}^{s}+\frac{R_{22}^{s}}{2}\right)\left(2R_{33}^{s}+R_{32}^{s}+R_{23}^{s}+\frac{R_{22}^{s}}{2}\right)\right]}{\left(2R_{11}+R_{12}+R_{21}+\frac{R_{22}}{2}\right)\left(2R_{33}+R_{32}+R_{23}+\frac{R_{22}}{2}\right)}+ \\ 2\frac{\sum_{s=1}^{k} \left[\left(\frac{R_{22}^{s}}{2}\right)-\left(2R_{13}^{s}+R_{12}^{s}+R_{23}^{s}+\frac{R_{22}^{s}}{2}\right)\left(2R_{31}^{s}+R_{32}^{s}+R_{21}^{s}+\frac{R_{22}^{s}}{2}\right)\right]}{\left(2R_{13}+R_{12}+R_{23}+\frac{R_{22}}{2}\right)\left(2R_{31}+R_{32}+R_{21}+\frac{R_{22}}{2}\right)}- \\ 2\frac{\sum_{s=1}^{k} \left[\left(2R_{12}^{s}+\frac{R_{22}^{s}}{2}\right)-\left(2R_{11}^{s}+R_{12}^{s}+R_{21}^{s}+\frac{R_{22}^{s}}{2}\right)\left(2R_{13}^{s}+R_{12}^{s}+R_{23}^{s}+\frac{R_{22}^{s}}{2}\right)\right]}{\left(2R_{11}+R_{12}+R_{21}+\frac{R_{22}}{2}\right)\left(2R_{13}+R_{12}+R_{23}+\frac{R_{22}}{2}\right)}- \end{matrix}$$

$$\begin{matrix} 2\frac{\sum_{s=1}^{k} \left[\left(2R_{21}^{s}+\frac{R_{22}^{s}}{2}\right)-\left(2R_{11}^{s}+R_{12}^{s}+R_{21}^{s}+\frac{R_{22}^{s}}{2}\right)\left(2R_{31}^{s}+R_{32}^{s}+R_{21}^{s}+\frac{R_{22}^{s}}{2}\right)\right]}{\left(2R_{11}+R_{12}+R_{21}+\frac{R_{22}}{2}\right)\left(2R_{31}+R_{32}+R_{21}+\frac{R_{22}}{2}\right)} \\ 2\frac{\sum_{s=1}^{k} \left[\left(2R_{23}^{s}+\frac{R_{22}^{s}}{2}\right)-\left(2R_{33}^{s}+R_{32}^{s}+R_{23}^{s}+\frac{R_{22}^{s}}{2}\right)\left(2R_{13}^{s}+R_{12}^{s}+R_{23}^{s}+\frac{R_{22}^{s}}{2}\right)\right]}{\left(2R_{33}+R_{32}+R_{23}+\frac{R_{22}}{2}\right)\left(2R_{13}+R_{12}+R_{23}+\frac{R_{22}}{2}\right)}- \\ 2\frac{\sum_{s=1}^{k} \left[\left(2R_{32}^{s}+\frac{R_{22}^{s}}{2}\right)-\left(2R_{33}^{s}+R_{32}^{s}+R_{23}^{s}+\frac{R_{22}^{s}}{2}\right)\left(2R_{31}^{s}+R_{32}^{s}+R_{21}^{s}+\frac{R_{22}^{s}}{2}\right)\right]}{\left(2R_{33}+R_{32}+R_{23}+\frac{R_{22}}{2}\right)\left(2R_{31}+R_{32}+R_{21}+\frac{R_{22}}{2}\right)} \end{matrix}$$

(A8)

By the same derivation of Equation (A8), $\text{Cov}(\hat{\beta}_{2},\hat{\beta}_{2})$ can be calculated as:

$$\begin{matrix} \text{Cov}\left(\widehat{\beta}_{2},\text{ }\widehat{\beta}_{2}\right) \\ =\text{Cov}\left\{\log\left[\begin{matrix} \frac{\left(4\left(R_{11}-N_{11}\right)+2\left(R_{12}-N_{12}\right)+2\left(R_{21}-N_{21}\right)+\left(R_{22}-N_{22}\right)\right)}{\left(4\left(R_{13}-N_{13}\right)+2\left(R_{12}-N_{12}\right)+2\left(R_{23}-N_{23}\right)+\left(R_{22}-N_{22}\right)\right)}\times\\ \frac{\left(4\left(R_{33}-N_{33}\right)+2\left(R_{32}-N_{32}\right)+2\left(R_{23}-N_{23}\right)+\left(R_{22}-N_{22}\right)\right)}{\left(4\left(R_{31}-N_{31}\right)+2\left(R_{32}-N_{32}\right)+2\left(R_{21}-N_{21}\right)+\left(R_{22}-N_{22}\right)\right)} \end{matrix}\right],\text{ }\right. \\ \text{ }\left.\log\left[\begin{matrix} \frac{\left(4\left(R_{11}-N_{11}\right)+2\left(R_{12}-N_{12}\right)+2\left(R_{21}-N_{21}\right)+\left(R_{22}-N_{22}\right)\right)}{\left(4\left(R_{13}-N_{13}\right)+2\left(R_{12}-N_{12}\right)+2\left(R_{23}-N_{23}\right)+\left(R_{22}-N_{22}\right)\right)}\times\\ \frac{\left(4\left(R_{33}-N_{33}\right)+2\left(R_{32}-N_{32}\right)+2\left(R_{23}-N_{23}\right)+\left(R_{22}-N_{22}\right)\right)}{\left(4\left(R_{31}-N_{31}\right)+2\left(R_{32}-N_{32}\right)+2\left(R_{21}-N_{21}\right)+\left(R_{22}-N_{22}\right)\right)} \end{matrix}\right]\right\} \end{matrix}$$

$$\begin{matrix} \text{= Cov}\left\{\log\left[4\left(R_{11}-N_{11}\right)+2\left(R_{12}-N_{12}\right)+2\left(R_{21}-N_{21}\right)+\left(R_{22}-N_{22}\right)\right]\right.+ \\ \text{ log}\left[4\left(R_{33}-N_{33}\right)+2\left(R_{32}-N_{32}\right)+2\left(R_{23}-N_{23}\right)+\left(R_{22}-N_{22}\right)\right]- \\ \text{ log}\left[4\left(R_{13}-N_{13}\right)+2\left(R_{12}-N_{12}\right)+2\left(R_{23}-N_{23}\right)+\left(R_{22}-N_{22}\right)\right]- \\ \text{ log}\left[4\left(R_{31}-N_{31}\right)+2\left(R_{32}-N_{32}\right)+2\left(R_{21}-N_{21}\right)+\left(R_{22}-N_{22}\right)\right],\text{ } \\ \text{ }\log\left[4\left(R_{11}-N_{11}\right)+2\left(R_{12}-N_{12}\right)+2\left(R_{21}-N_{21}\right)+\left(R_{22}-N_{22}\right)\right]+ \\ \text{ log}\left[4\left(R_{33}-N_{33}\right)+2\left(R_{32}-N_{32}\right)+2\left(R_{23}-N_{23}\right)+\left(R_{22}-N_{22}\right)\right]- \\ \text{ log}\left[4\left(R_{13}-N_{13}\right)+2\left(R_{12}-N_{12}\right)+2\left(R_{23}-N_{23}\right)+\left(R_{22}-N_{22}\right)\right]- \\ \text{ }\left.\text{ log}\left[4\left(R_{31}-N_{31}\right)+2\left(R_{32}-N_{32}\right)+2\left(R_{21}-N_{21}\right)+\left(R_{22}-N_{22}\right)\right]\right\} \end{matrix}$$

(A9)

The first item in Equation (A9) is calculated as

$$\begin{matrix} \text{Cov}\left\{\log\left[4\left(R_{11}-N_{11}\right)+2\left(R_{12}-N_{12}\right)+2\left(R_{21}-N_{21}\right)+\left(R_{22}-N_{22}\right)\right],\text{ }\right. \\ \text{ }\left.\log\left[4\left(R_{11}-N_{11}\right)+2\left(R_{12}-N_{12}\right)+2\left(R_{21}-N_{21}\right)+\left(R_{22}-N_{22}\right)\right]\right\} \\ \approx\frac{1}{4\text{E}\left(R_{11}-N_{11}\right)+2\text{E}\left(R_{12}-N_{12}\right)+2\text{E}\left(R_{21}-N_{21}\right)+\text{E}\left(R_{22}-N_{22}\right)}\times\\ \text{ }\frac{1}{4\text{E}\left(R_{11}-N_{11}\right)+2\text{E}\left(R_{12}-N_{12}\right)+2\text{E}\left(R_{21}-N_{21}\right)+\text{E}\left(R_{22}-N_{22}\right)}\times\\ \text{ Cov}\left[4\left(R_{11}-N_{11}\right)+2\left(R_{12}-N_{12}\right)+2\left(R_{21}-N_{21}\right)+\left(R_{22}-N_{22}\right)\right.,\text{ } \\ \left.\text{ }4\left(R_{11}-N_{11}\right)+2\left(R_{12}-N_{12}\right)+2\left(R_{21}-N_{21}\right)+\left(R_{22}-N_{22}\right)\right]\text{ } \end{matrix}$$

$$\begin{matrix} =\frac{1}{4\text{E}\left(R_{11}-\sum_{s=1}^{k} N_{11}^{s}\right)+2\text{E}\left(R_{12}-\sum_{s=1}^{k} N_{12}^{s}\right)+2\text{E}\left(R_{21}-\sum_{s=1}^{k} N_{21}^{s}\right)+\text{E}\left(R_{22}-\sum_{s=1}^{k} N_{22}^{s}\right)}\times\\ \text{ }\frac{1}{4\text{E}\left(R_{11}-\sum_{s=1}^{k} N_{11}^{s}\right)+2\text{E}\left(R_{12}-\sum_{s=1}^{k} N_{12}^{s}\right)+2\text{E}\left(R_{21}-\sum_{s=1}^{k} N_{21}^{s}\right)+\text{E}\left(R_{22}-\sum_{s=1}^{k} N_{22}^{s}\right)}\times\\ \text{ Cov}\left[4\sum_{s=1}^{k} N_{11}^{s}+2\sum_{s=1}^{k} N_{12}^{s}+2\sum_{s=1}^{k} N_{21}^{s}+\sum_{s=1}^{k} N_{22}^{s},\text{ }4\sum_{s=1}^{k} N_{11}^{s}+2\sum_{s=1}^{k} N_{12}^{s}+2\sum_{s=1}^{k} N_{21}^{s}+\sum_{s=1}^{k} N_{22}^{s}\right] \end{matrix}$$

(A10)

Based on the hypergeometric distribution assumption, we have

$$\text{E}\left(R_{ij}-\sum_{s=1}^{k} N_{ij}^{s}\right)=R_{ij}-\text{E}\left(\sum_{s=1}^{k} N_{ij}^{s}\right)=R_{ij}-\frac{R_{ij}}{2}=\frac{R_{ij}}{2}=\text{E}\left(\sum_{s=1}^{k} N_{ij}^{s}\right)$$

Then, Equation (A10) will be equal to Equation (A5). We can extend this result to all items in Equation (A9), and we can conclude $\text{Var}\left(\widehat{\beta}_{1}\right)=\text{Var}\left(\widehat{\beta}_{2}\right)$.

For the last item in Equation (A3),

$$\begin{matrix} \text{Cov}\left(\widehat{\beta}_{1},\text{ }\widehat{\beta}_{2}\right) \\ =\text{Cov}\left\{\log\left[\frac{(4N_{11}+2N_{12}+2N_{21}+N_{22})(4N_{33}+2N_{32}+2N_{23}+N_{22})}{(4N_{13}+2N_{12}+2N_{23}+N_{22})(4N_{31}+2N_{32}+2N_{21}+N_{22})}\right],\right.\text{ } \\ \text{ }\left.\log\left[\begin{matrix} \frac{\left(4\left(R_{11}-N_{11}\right)+2\left(R_{12}-N_{12}\right)+2\left(R_{21}-N_{21}\right)+\left(R_{22}-N_{22}\right)\right)}{\left(4\left(R_{13}-N_{13}\right)+2\left(R_{12}-N_{12}\right)+2\left(R_{23}-N_{23}\right)+\left(R_{22}-N_{22}\right)\right)}\times\\ \frac{\left(4\left(R_{33}-N_{33}\right)+2\left(R_{32}-N_{32}\right)+2\left(R_{23}-N_{23}\right)+\left(R_{22}-N_{22}\right)\right)}{\left(4\left(R_{31}-N_{31}\right)+2\left(R_{32}-N_{32}\right)+2\left(R_{21}-N_{21}\right)+\left(R_{22}-N_{22}\right)\right)} \end{matrix}\right]\right\} \end{matrix}$$

$$\begin{matrix} =\text{Cov}\left\{\log(4N_{11}+2N_{12}+2N_{21}+N_{22})+\log(4N_{33}+2N_{32}+2N_{23}+N_{22})\right.- \\ \text{ log}(4N_{13}+2N_{12}+2N_{23}+N_{22})-\log(4N_{31}+2N_{32}+2N_{21}+N_{22}),\text{ } \\ \text{ l}\mathrm{og}\left[4\left(R_{11}-N_{11}\right)+2\left(R_{12}-N_{12}\right)+2\left(R_{21}-N_{21}\right)+\left(R_{22}-N_{22}\right)\right]+ \\ \text{ log}\left[4\left(R_{33}-N_{33}\right)+2\left(R_{32}-N_{32}\right)+2\left(R_{23}-N_{23}\right)+\left(R_{22}-N_{22}\right)\right]- \\ \text{ log}\left[4\left(R_{13}-N_{13}\right)+2\left(R_{12}-N_{12}\right)+2\left(R_{23}-N_{23}\right)+\left(R_{22}-N_{22}\right)\right]- \\ \text{ }\left.\text{ log}\left[4\left(R_{31}-N_{31}\right)+2\left(R_{32}-N_{32}\right)+2\left(R_{21}-N_{21}\right)+\left(R_{22}-N_{22}\right)\right]\right\} \end{matrix}$$

(A11)

The first item in Equation (A11) is calculated as:

$$\begin{matrix} \text{Cov}\left\{\text{l}\mathrm{og}(4N_{11}+2N_{12}+2N_{21}+N_{22}),\text{ }\right. \\ \left.\text{ l}\mathrm{og}\left[4\left(R_{11}-N_{11}\right)+2\left(R_{12}-N_{12}\right)+2\left(R_{21}-N_{21}\right)+\left(R_{22}-N_{22}\right)\right]\right\} \\ \approx\frac{1}{4\text{E}(N_{11})+2\text{E(}N_{12})+2\text{E(}N_{21})+\text{E(}N_{22})}\times\\ \text{ }\frac{1}{4\text{E}\left(R_{11}-N_{11}\right)+2\text{E}\left(R_{12}-N_{12}\right)+2\text{E}\left(R_{21}-N_{21}\right)+\text{E}\left(R_{22}-N_{22}\right)}\times\\ \text{ Cov}\left[4N_{11}+2N_{12}+2N_{21}+N_{22}\right.,\text{ } \\ \left.\text{ }4\left(R_{11}-N_{11}\right)+2\left(R_{12}-N_{12}\right)+2\left(R_{21}-N_{21}\right)+\left(R_{22}-N_{22}\right)\right] \\ =\frac{1}{4\text{E}(N_{11})+2\text{E(}N_{12})+2\text{E(}N_{21})+\text{E(}N_{22})}\frac{1}{4\text{E}(N_{11})+2\text{E(}N_{12})+2\text{E(}N_{21})+\text{E(}N_{22})}\times\\ \text{ }-\text{Cov}\left[4N_{11}+2N_{12}+2N_{21}+N_{22},\text{ }4N_{11}+2N_{12}+2N_{21}+N_{22}\right] \\ =-\text{ Cov}\left[\log(4N_{11}+2N_{12}+2N_{21}+N_{22}),\text{ log(4}N_{11}+2N_{12}+2N_{21}+N_{22})\right] \end{matrix}$$

(A12)

By calculation, we will find each item in the expansion of Equation (A11) has simply an opposite sign of each item in the expansion of Equation (A4). Thus, we will have

$$\text{Cov}\left(\widehat{\beta}_{1},\text{ }\widehat{\beta}_{2}\right)=-\text{Var}\left(\widehat{\beta}_{1}\right)=-\text{Var}\left(\widehat{\beta}_{2}\right)$$

Finally, we can find that $\mathrm{Var}\left( \hat{\beta}_{1}-\hat{\beta}_{2} \right)=4\mathrm{Var}\left( \hat{\beta}_{1} \right) \left[ \mathrm{or} 4\mathrm{Var}\left( \hat{\beta}_{2} \right) \right]$.
